# Prescription Department

**Published:** 1894-07

**Authors:** 


MYALGIA.

Dr. Augustus A. Eshner, in the Philadelphia *Polyclinic*, states that he has found the following formula of service:

℞ Tinct. guaiac. ammoniat.,

Ext. cimicifugæ fld.,

Ext. erythroxyli fld., āā f ʒ j.

M. Sig. A teaspoonful is to be taken three times a day before meals.

When constipation co-exists an equal proportion of fluid extract of cascara is added.

FERMENTATIVE DYSPEPSIA.

The *Columbus Medical Journal* selects the following prescription from the Asclepiad:

℞ Ol. creasoti pur., *m* xij.

Sp. tennuris, ℥ iiss,

Ammon. benzoat., ʒ ij.

Glycerin. pur., ʒ vj.

Infus. caryophylli, ℥ vj.

M. Sig. Tablespoonful two or three times a day, between meals, in water.

CRACKED NIPPLES.

M. Lepage uses compresses soaked in the following solution:

℞ Red iodide of mercury, gr. iss-iij.
Alcohol, ℥ iss.
Distilled water, ℥ xivss.
Glycerin. ℥ xvj.—M.

—*Universal Med. Journal.*

APPLICATION FOR CHRONIC ULCER.

℞ Acid. chromic., gr. xxx.
Acid. tannic., gr. xx.
Morph. sulph., gr. v.
Chloral. hydrat., ʒ vj.
Aquæ, Oj.

M. Sig. Apply frequently, during the day, with a camel-hair pencil; in the interim keep the ulcer covered lightly with a cloth or bandage.—*Ibid.*

INFLAMMATORY EARACHE.

℞ Powered menthol, gr. xx.
Camphor, gr. xx.
Vaselin, ʒ vj.

M. ft. ungt.—*Ibid.*

CHRONIC CYSTITIS.

℞ Tr. collinsoniæ, ʒ vi.

Copaibæ, ʒ iij.

Liq. morphinæ, ʒ ss.

Liq. potassæ ℥ ss.

Ol. menth. pip., ♏ iij.

Aq. camphoræ, q. s. ad. ℥ vi.

M. Sig. A teaspoonful to be taken every four hours.—*Dr. Chevers, in Med. Press and Cir.*

VOMITING OF PREGNANCY.

For stubborn cases, Dr. Julian Berry of Mace, Ind., recommends, in the *Memphis Medical Journal:*

℞ Fld. ext. valerian, ℥ j.

Fowler's sol. arsenic, ♏ xvj.

Sod. bicarb., ʒ j.

M. Sig. Teaspoonful every two or three hours.

PAINFUL DENTITION.

℞ Muriate of cocaine, gr. iss.
Tincture of conium,
Syrup, āā ʒ ij.

M. Sig. Rub on the gums several times daily.—*N. Y. Polyclinic.*

A CARBONATED LAXATIVE.

℞ Sodii phosphate, ℥ j.

Aquæ destil., f ℥ x.

Syrup simplicis, f ℥ ij.

Tinct. limonis, gtt. xxv.

Acid citric, } āā ʒ ss.

Sodii bicarbonate, }

M. Sig. Two tablespoonfuls, or more, as required.—*Paul, Les Nouv. Rem., Med. News.*

TYPHOID FEVER.

When the skin is dry and the lips parched, I often use the following mixture:

℞ Potass. chlorat., ʒ ss.

Sp. æth. nit., ʒ vj.

Liq. ammon. ac., q. s. ad. ℥ iij.

M. Sig; ʒ ij. every three or four hours.—*Dr. R. B. Norment, of Baltimore, in the St. Louis Clinique.*

